# A prospective analysis to assess the multifactorial risk of childhood-onset hypertension: the ExAMIN Youth SA study

**DOI:** 10.1038/s41440-025-02309-6

**Published:** 2025-08-15

**Authors:** Chanelle Volschenk, Esmé Jansen van Vuren, Annemarie Wentzel, Ruan Kruger

**Affiliations:** 1 Hypertension in Africa Research Team (HART), North-West University, Potchefstroom, South Africa; 2 South African Medical Research Council Unit for Hypertension and Cardiovascular Disease, North-West University, Potchefstroom, South Africa

**Keywords:** Children, risk factors, cardiovascular health, South Africa, implemental hypertension

## Abstract

Childhood-onset hypertension tracks into adulthood and is on the rise globally. Identifying risk factors in early childhood remains of epidemiological importance for developing early intervention and prevention strategies to mitigate premature hypertension onset. This study explored the changes in blood pressure and the predictive value of individual and composite baseline risk factors for elevated blood pressure over a 4-year period in South African children. We included 767 healthy children (aged 5–9 years at baseline) with a mean follow-up time of four years. Office blood pressure, anthropometry, cardiorespiratory fitness, health-related quality of life, food intake and urinary biomarkers were measured. Children were stratified by blood pressure status according to the 2017 American Academy of Pediatrics guidelines. Individual baseline risk factors as well as composite risk factors were assessed to predict follow-up blood pressure status. The prevalence of elevated blood pressure declined by 6% over four years. Longitudinally, age (HR:1.78; *p* = 0.005), ethnicity (HR:0.048; *p* = 0.001), socioeconomic status (HR:0.42; *p* = 0.004) and sugar-sweetened beverages intake (HR:1.67; *p* = 0.026) predicted elevated blood pressure over four years. No significant results were observed with composite risk factors cross-sectionally, however factor pattern 1 (socioeconomic status, family history, meat and milk product intake) indicated a lower risk of elevated blood pressure at follow-up (HR:0.74; *p* = 0.042). Multiple risk factors, including diet and socioeconomic status, contribute to elevated blood pressure in South African children. Early multifaceted interventions targeting these factors are essential to prevent long-term cardiovascular disease.

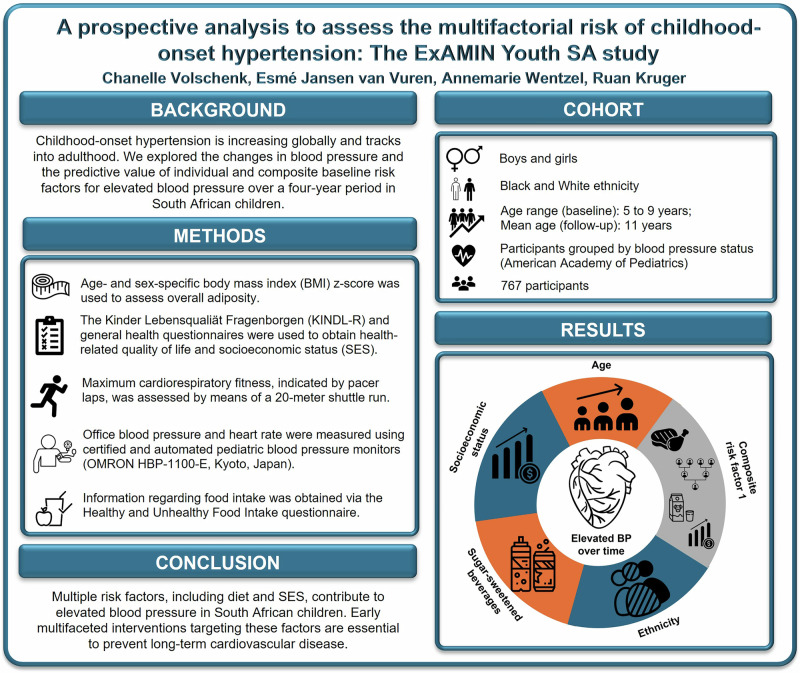

## Introduction

Primary hypertension in childhood is becoming a concern since abnormal blood pressure (BP) during childhood will most likely remain prevalent or even worsen over the life course [1–3]. The prevalence of childhood-onset primary hypertension is rising globally [4] and is more commonly reported than secondary hypertension [5–7] as it can be ascribed mainly to the increase in the prevalence of childhood overweight/obesity (OW/OB) as observed in Southern Africa [8–10]. Recent evidence indicated that the prevalence of elevated BP in African children was reported to be between 2.7% and 50.5% [9, 11]. The large variation seems to be influenced by several methodological challenges, the greatest being a lack of normal BP reference values for children in sub-Saharan Africa. Additionally, the prevalence of OW/OB is lower in the Eastern and Western regions of Africa which implies alternative causes of hypertension other than increased adiposity [11]. Thus, with such a high prevalence of hypertension and elevated BP amongst children and adolescents in Africa, distinct causes should be identified and studied to develop early intervention and prevention strategies to lower the burden of cardiovascular diseases (CVDs) in the growing adult population.

Several different modifiable and non-modifiable risk factors have been identified as contributing factors for hypertension development in adults as well as children [12]. Various cross-sectional studies in Africa have reported on individual risk factors which are associated with elevated BP in children [12–22]. The primary focus in these aforementioned pediatric studies was OW/OB [19, 23, 24] while other individual risk factors included healthy and unhealthy food intake [25–27], physical activity and fitness [18, 28], socioeconomic status (SES) [29] and birth weight [30–33]. Results from a prospective study including participants from the United States, Finland and Australia have indicated that when traditional cardiovascular risk factors (body mass index (BMI), total cholesterol level, triglycerides, systolic blood pressure (SBP) and youth smoking) in children aged between 3 and 19 years were combined as a composite score, there was higher risk associated with cardiovascular events in midlife compared to the individual risk factors [34]. These results obtained from countries in the global North cannot be extrapolated to countries in sub-Saharan Africa mainly because of sociodemographic differences, thus the factors that contribute to hypertension in sub-Saharan Africa may be different. Longitudinal studies in sub-Saharan Africa are scarce, hindering the investigation of cardiovascular risk factors over time. Limited evidence exists for a Southern African-specific cardiovascular risk factor profile, leaving the relationship between these factors and high BP later in life largely unknown.

Identifying risk factors for childhood hypertension or changes in blood pressure before adulthood is important because elevated blood pressure in children often persists into adulthood, increasing the risk of long-term cardiovascular disease and organ injury. Early detection allows timely intervention through lifestyle modifications—such as improving diet, increasing physical activity, and managing weight—that can prevent or delay the development of sustained hypertension and its serious health consequences. Furthermore, recognizing both modifiable (e.g., obesity, salt intake, sedentary lifestyle) and non-modifiable (e.g., family history, low birth weight) risk factors in childhood enable targeted screening and monitoring, improving cardiovascular outcomes across the lifespan. Herewith, we aimed to investigate changes in BP and the predictive value of individual risk factors as well as composite risk factor patterns on incident elevated BP of prepubescent children living in South Africa over a four-year follow-up period.

## Methods

### Study design and demographics

This study forms part of the Exercise, Arterial Modulation and Nutrition in Youth South Africa (ExAMIN Youth SA) study [35]. The baseline phase of the ExAMIN Youth SA study was conducted between 2017 and 2019 and the follow-up phase between 2021 and 2022. The mean follow-up time was four years. The original study included healthy children of both sexes (aged between five and nine years) attending public primary schools in Potchefstroom and Klerksdorp in the North West province of South Africa. We made use of the South African School Quintile System which is managed by the Department of Basic Education in South Africa. This system allocates funding and resources fairly across public schools based on the surrounding community’s socioeconomic status. This system allocates a specific quintile to each school where quintile 1 represents the poorest, quintile 2 and 3 – poor to lower-middle income, quintile 4 – middle income and finally quintile 5 – least poor. For this study the recruitment took place in quintile 3 to quintile 5 schools with a goal to capture the broad spectrum of low-to-middle income learners which may be representative of much of the South African public school-going population. Further in detail participant recruitment was explained elsewhere [35]. For both baseline and follow-up, written informed consent (children older than seven years) or assent (children younger than seven years) as well as parental permission were obtained before any data collection took place. The only exclusion criteria for this study were children who were unwilling to participate.

**Fig. 1:**
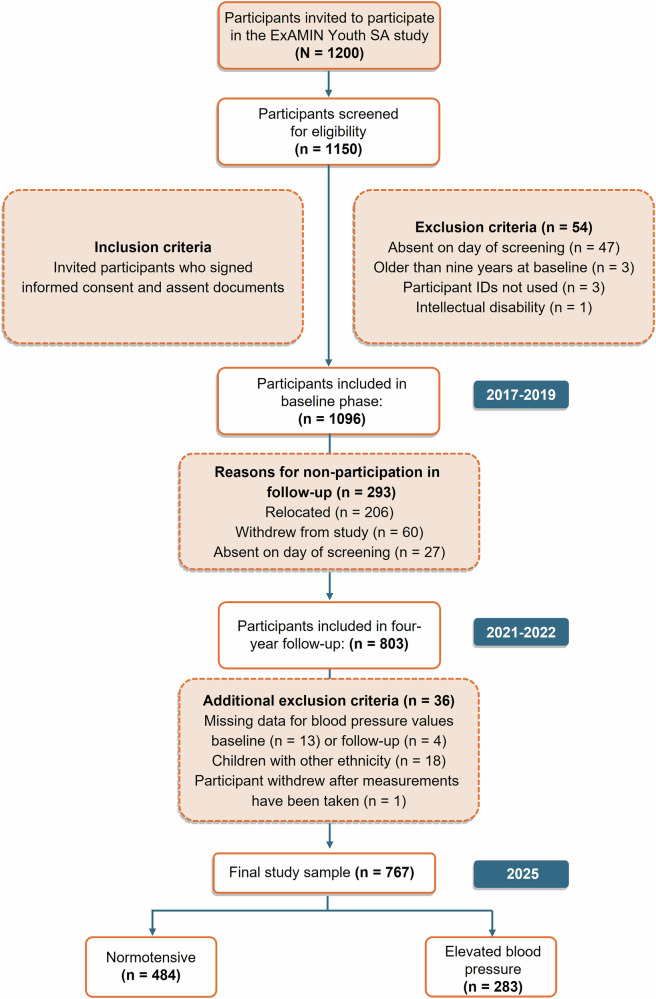
Consort diagram of the current study

The four-year follow-up phase of the ExAMIN Youth SA study had a participation success rate of 73.3% (*n* = 803 participants). Our current study population comprised 767 participants as indicated by Fig. 1. This study was approved by the Health Research Ethics Committee of the North-West University (NWU-00091-16-A1) and registered at ClinicalTrials.gov (NCT04056377).

### General health and demographic questionnaire

Parents or caregivers were asked to supply the required information by making use of a standard General Health and Demographic Questionnaire to collect socio-demographic information, including personal (age, sex and ethnicity) and family information (e.g., parents’ education, occupation, type of home and household income). For SES, a composite score was calculated using responses from the General Health and Demographic questionnaire, which included the means of both parents’ household income, education level, and employment status. This summative score was calculated by the sum of the means, where higher scores indicated a higher SES [36].

### Food intake questionnaire

Dietary intake was evaluated by using an unquantified food group frequency questionnaire which was completed by parents or caregivers. This validated healthy and unhealthy food intake questionnaire [37] was based on prior questionnaires that assessed these food intake categories. The questionnaire was designed to collect information on the frequency of consumption of healthy and unhealthy food types. The food groups included in this questionnaire reflected foods reported to be eaten by South African school children, and they included four groups of healthy foods (fruit, vegetables, milk, meat/fish/poultry/eggs) and six groups of unhealthy foods (hot and cold sugar-sweetened beverages, candy, salty snacks, cookies and cakes, and fast foods). To facilitate responses, a colored picture file with food examples from each group was shown alongside the questionnaire. The mean frequency of consumption for each food category was computed, and food group frequencies were reported in categorized format.

### Health-related quality of life questionnaire

The Kinder Lebensqualiät Fragenborgen (KINDL-R) questionnaire was used to obtain information regarding health-related quality of life of the participants [38]. The KINDL-R has previously been validated in a South African context [39]. The questionnaire consists of seven subdomains (positive affect emotional well-being, negative affect emotional well-being, positive everyday functioning, negative everyday functioning, physical well-being, friends and self-esteem). Each question was rated on a 5-point Likert scale. A conglomeration of all seven subdomains forms the composite total quality of life score, where a higher score reflects a better quality of life. The following formula was used to calculate the 100 variables for each subdomain:

$${{{\rm{subdomain}}}}_{100}=\frac{\left({\mbox{sum of items in subdomain}}\right)\,-{\mbox{lowest possible score}}}{{\mbox{possible range of raw score}}}\,{{\rm{X}}}\,100\,$$

### Anthropometric measurements

Anthropometric measurements comprised body weight (kg) and body height (cm). Body weight was determined using a SECA 813 digital scale to the closest 0.1 kg. Body height was measured using a SECA 213 stadiometer (Birmingham, United Kingdom) without shoes and with the perpendicular board to the nearest 0.1 cm. Age- and sex-adjusted BMI z-scores calculated according to World Health Organization guidelines [40] were used for assessment of overall adiposity in children.

### Cardiorespiratory fitness and physical activity levels

A 20-meter shuttle run was performed to assess maximum cardiorespiratory fitness (CRF). Cardiorespiratory fitness was measured in the morning during school hours, with the same equipment and research team at each participating school. Following a five-minute warm-up period, the well-established and confirmed CRF test was administered [41]. In this continuous endurance test, children were required to run back and forth across a 20-meter distance at an initial speed of 8.0 km/h. Each minute, the speed was increased by 0.5 km/h, accompanied by a bleep audio sound. When the participant did not cross the 20 m line at the time of the bleep sound in two consecutive 20 m attempts, he or she had reached the individual maximum of high-intensity exercise. Throughout each assessment, all participants were verbally encouraged to perform to their full potential. The score was based on the number of stages (1 stage = 1 minute) completed with a precision of 0.5 stages.

### Blood pressure

To limit inter-observer variability, office BP was taken with a certified and automated pediatric BP monitor (OMRON HBP-1100-E, Kyoto, Japan). Each participant was equipped with the appropriately sized cuff for measurement. Before taking their BP, the participant was asked to sit calmly for five minutes before the measurement whereafter they were asked to sit with their back supported, feet flat on the floor, and right arm supported at heart level. Blood pressure was measured five times consecutively, with a one-minute rest period in between. The mean of the three measurements with the least deviation was utilized for further analyses [19]. Children were stratified into BP status groups according to the 2017 clinical practice guidelines for screening and management of high BP in children published by the American Academy of Pediatrics (AAP) [42].

### Urinalyses

Early on the day of participation in the study, participants were requested to provide a midstream urine sample in the privacy of their own home, with the help of their parents. The participants were given sealable urine containers to collect their first urine samples on the day of the study. All samples were processed and aliquoted into cryovials, whereafter samples were stored in bio-freezers at -80°C until analysis. We used urine samples to obtain urinary sodium (mmol/L) and urinary potassium (mmol/L). The urinary sodium-to-potassium-ratio (Na^+^/K^+^-ratio) was calculated by dividing creatinine-corrected urinary sodium by creatinine-corrected urinary potassium [37].

### Statistical analyses

Statistical analyses were performed with IBM^®^ SPSS^®^ Statistics version 30 software (IBM Corporation; Armonk, New York, USA). GraphPad Prism version 5.03 (GraphPad Software Inc., CA, USA) was used for the graphical illustration of data. Participants were stratified according to BP status as described by the AAP. For baseline data analyses, we used Chi-square tests for dichotomous variables and independent T-tests and ANCOVAs for continuous variables for comparisons between BP groups. For follow-up comparisons, dependent T-tests (continuous variables) and McNemar testing (dichotomous variables) were used. The dimension reduction function of SPSS was used to identify risk factor patterns. Principal component analysis was used and factors with an Eigenvalue of >1.5 were retained. The Oblimin rotation method was used to obtain independent factors. A factor loading ≥0.5 was included to interpret the factor patterns that were automatically calculated by the statistical software and used in further analyses. Backward stepwise multiple regression analyses were used to identify independent associations of BP with the individual risk factors and composite risk factor patterns at baseline only. Odds ratios were used to identify the likelihood of elevated BP at baseline. Hazard ratios were used with the different individual baseline risk factors as well as composite risk factor clusters at baseline as predictors to identify the relative risk for developing incident elevated BP at follow-up.

## Results

### General characteristics of participants for baseline and follow-up

**Fig. 2:**
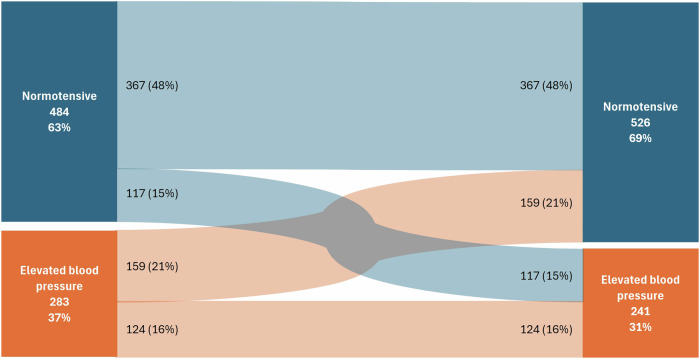
Four-year change in blood pressure status from baseline to follow-up

The baseline characteristics of the participants stratified by BP status are reported in Table S1. The normotensive group comprised 484 (63%) children, while the elevated BP group comprised 283 (37%) children. Figure 2 shows the changes in BP status from baseline to follow-up. From the children who were normotensive at baseline, 48% (*n* = 367) remained normotensive at follow-up, while 15% (*n* = 117) developed elevated BP after the four-year follow-up period. Children whose BP normalized from baseline to follow-up were 21% (*n* = 159), while 16% (*n* = 124) of children remained in the elevated BP group from baseline to follow-up.

Basic characteristics of the baseline and follow-up phases of this study were reported in Table 1 stratified by baseline BP status. Independent of stature growth (body height), in the normotensive BP group, it was evident that mean SBP increased with 9 mmHg and mean diastolic blood pressure (DBP) increased with 5 mmHg after a four-year follow-up period (all *p* < 0.001). In the elevated BP group, SBP, DBP and MAP did not significantly differ from baseline to follow-up. From baseline to follow-up, 11% (*n* = 85) of the children became OW/OB (normotensive group; *n* = 40 and elevated BP group; *n* = 23; all *p* < 0.001). Over the follow-up period, SES and CRF improved in both BP groups while health-related quality of life declined significantly over time (all *p* < 0.017). In both the normotensive and elevated BP groups, the intake of sugar-sweetened beverages declined from baseline to follow-up (normotensive: *p* = 0.002; elevated BP: *p* < 0.001) while fast-food intake increased (normotensive: *p* = 0.005; elevated BP: *p* = 0.029). Additionally, we also found that in the elevated BP group, vegetable (*p* = 0.015) and meat product consumption (*p* = 0.038) increased over the four-year period. In the normotensive group, urinary sodium and potassium levels have also declined from baseline to follow-up in this group leading to a decreased sodium-potassium-ratio (all *p* < 0.001). In the elevated BP group, a significant decrease was only evident in urinary sodium (*p* < 0.001) and not urinary potassium (*p* = 0.20), albeit the sodium-potassium-ratio also declined significantly (*p* < 0.001) from baseline to follow-up.

**Table 1:** Changes in population characteristics between baseline and four-year follow-up stratified by baseline blood pressure status

	Normal blood pressure (*n* = 484)				Elevated blood pressure (*n* = 283)		
	Baseline; Follow-up	Difference		p-value	Baseline; Follow-up	Difference	p-value
**Non-modifiable risk factors**							
Age, years	7; 11		+4	**<0.001**	8; 11	+3	**<0.001**
Socioeconomic status	5.76; 5.96		+0.20	**0.011**	6.03; 6.27	+0.24	**<0.001**
Family history, yes	68; 64		−4	0.17	49; 46	−3	0.22
Health-related quality of life	60.1; 40.1		−20.0	**<0.001**	61.2; 41.4	−19.8	**<0.001**
**Modifiable risk factors**							
**Blood pressure**							
Systolic blood pressure, mmHg^a^	97; 106		+9	**<0.001**	110; 111	+1	0.37
Diastolic blood pressure, mmHg^a^	61; 66		+5	**<0.001**	70; 69	−1	0.24
Mean arterial pressure, mmHg^a^	76; 82		+6	**<0.001**	87; 87	─	0.80
***Body composition***							
Body weight, kg	23.9; 38.6		+14.4	**<0.001**	26.5; 42.5	+16.0	**<0.001**
Body height, cm	122.1; 144.2		+22.1	**<0.001**	123.8; 144.9	+21.1	**<0.001**
BMI-z score	−0.17; 0.01		+0.18	**<0.001**	0.32; 0.42	+0.10	**0.004**
**Adiposity status**							
Normal weight	427; 387		−40	**<0.001**	198; 175	−23	**<0.001**
Overweight/Obese	57; 97		+40	**<0.001**	85; 108	+23	**<0.001**
***Cardiorespiratory fitness***							
20-m shuttle run, laps	28.6; 38.1		+9.5	**<0.001**	29.7; 39.0	+9.3	**<0.001**
Heart rate, bpm	86; 79		−7	**<0.001**	89; 80	−9	**<0.001**
***Food intake frequency per week***							
Fruits	3.21; 3.15		−0.06	0.63	3.29; 3.56	+0.27	0.092
Vegetables	3.05; 2.98		−0.07	0.56	2.83; 3.18	+0.35	**0.015**
Milk, yogurt	4.37; 4.09		−0.28	0.060	4.13; 3.99	−0.14	0.39
Meat, fish, poultry, eggs	4.82; 4.77		−0.05	0.70	4.72; 5.05	+0.33	**0.038**
Sugar-sweetened beverages	4.10; 3.64		−0.46	**0.002**	4.13; 3.35	−0.78	**<0.001**
Tea and hot drinks with sugar	3.25; 3.34		+0.09	0.58	3.19; 3.30	+0.11	0.59
Sweets	2.83; 2.93		+0.10	0.47	2.84; 2.62	−0.22	0.20
Salty snacks	2.85; 2.91		+0.06	0.67	3.06; 2.77	−0.29	0.10
Cookies, cakes, biscuits	2.09; 2.14		+0.05	0.70	2.01; 2.01	─	1.0
Fast foods	1.66; 1.95		+0.29	**0.005**	1.69; 2.01	+0.32	**0.029**
***Urinary biomarkers***							
Sodium	16.3; 12.5		−3.8	**<0.001**	16.6; 13.1	−3.5	**<0.001**
Potassium	3.46; 3.01		−0.45	**<0.001**	3.24; 3.03	−0.21	0.20
Sodium/Potassium ratio	0.58; 0.45		−0.13	**<0.001**	0.62; 0.49	−0.13	**<0.001**

Values were obtained using McNemar test, dependent T-tests and ANCOVA. Values are reported as means and mean change. A negative difference indicates a decline in values from baseline to follow-up. Data in boldface indicates statistical significance for *p* ≤ 0.05

^a^Blood pressure values were adjusted for body height and in instance of follow-up blood pressure, baseline blood pressure was also accounted for. Urinary biomarkers were corrected for urinary creatinine

### Cross sectional findings

Independent associations of baseline risk factors with baseline BP are reported in Table S2. In the total group, SBP was associated with age (β = 0.18; *p* = 0.014) and BMI-z score (β = 0.29; *p* < 0.001), while DBP associated with BMI-z score (β = 0.20; *p* = 0.002) and positive family history of CVD (β = 0.41; *p* = 0.008). In the normotensive group, SBP was associated with age (β = 0.14; *p* = 0.028) and BMI-z score (β = 0.16; *p* = 0.006), while in the elevated BP group, SBP was only associated with BMI-z score (β = 0.19; *p* = 0.034). Diastolic blood pressure was associated with age (β = 0.13; *p* = 0.040) in the normotensive group, while no associations were evident in the elevated BP group.

Odds ratios of baseline risk factors and baseline elevated BP are reported in Table S3. When looking at the probability of individual baseline risk factors (age, sex, ethnicity, SES, family history, sodium-potassium-ratio, fruits, vegetables, meat, milk, sugar-sweetened beverages, sugary hot drinks, sweets, salty snacks, cookies and cake, fast foods, BMI-z score, CRF and health-related quality of life) associating with elevated BP in children at baseline, we found that only BMI-z score (OR = 2.21; *p* < 0.001) associated with elevated BP at baseline. These results indicate that for every standard deviation increase in BMI-z score there is a two-fold increase in the odds of having elevated BP at baseline.

### Longitudinal findings

**Fig. 3:**
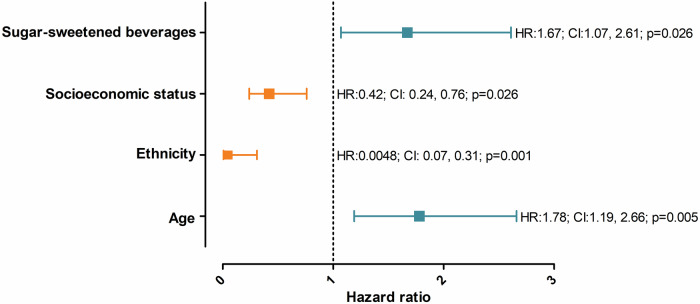
Relative risk of baseline risk factors predicting elevated blood pressure after a 4-year period. The whiskers report the 95% confidence intervals with the dots representing the hazard ratio

The relative risk of baseline risk factors predicting elevated BP over a four-year period were investigated and are reported in Fig. 3 and Table S4. We observed that an increase in age (HR:1.78; *p* = 0.005), Black ethnicity (HR:0.048; *p* = 0.001), lower SES (HR:0.42; *p* = 0.004) and higher intake of sugar-sweetened beverages (HR = 1.67; *p* = 0.026), were significant predictors of follow-up elevated BP.

### Composite risk factor patterns findings

Principal component analysis was done to investigate the potential of composite risk factor patterns associated with and predicting follow-up BP. The five factors that were obtained during the factor analyses are reported in Table S5. Multiple regression analyses were performed to determine the independent associations of baseline-identified risk factor patterns with baseline BP in the BP status groups (Table S6). Factors of interest were Factor 1 (SES, family history, meat and milk product consumption) and Factor 4 (BMI-z score and CRF).

In the total group, BP was inversely associated with Factor 1 (DBP: β = −0.16; *p* = 0.012) and Factor 4 (SBP: β = −0.15; *p* = 0.001 and DBP: β = −0.097 *p* = 0.040). In the normotensive group, SBP was inversely associated with Factor 1 (β = −0.16; *p* = 0.032), while both SBP and DBP were inversely associated with Factor 4 (SBP: β = −0.14; *p* < 0.001 and DBP: β = −0.14; *p* < 0.001). In the elevated BP group, SBP was inversely associated with Factor 4 (β = −0.14; *p* = 0.025), while DBP was inversely associated with Factor 1 (β = −0.24; *p* = 0.015).

The probability of risk factor patterns to increase the likelihood for elevated BP at baseline was reported as odds ratios in Table S7. No risk factor patterns were found to significantly predict elevated BP at baseline.

Table 2 reported the probability of risk factor patterns to develop elevated BP after a four-year period. Factor 1 significantly predicted a lower relative risk for having elevated BP after a four-year period (HR: 0.74; *p* = 0.042).

**Table 2:** Probability of baseline risk factors patterns in children predicting elevated blood pressure over a four-year-follow-up period

	Hazard ratio	95% CI	*p* value
Factor 1	0.74	0.55; 0.99	**0.042**
Factor 2	1.10	0.95; 1.27	0.20
Factor 3	0.98	0.85; 1.13	0.75
Factor 4	0.92	0.77; 1.09	0.34
Factor 5	0.90	0.76; 1.06	0.22

Cox-regression analyses were used to obtain values. Models consisted of age, sex, ethnicity, baseline blood pressure and a factor

## Discussion

In a cohort of apparently healthy children, we explored i) changes in BP and ii) the predictive value of individual risk factors as well as composite risk factor patterns on incident elevated BP over a four-year follow-up period. From baseline to follow up, 15% of children developed elevated BP, while 16% remained in the elevated BP group. At baseline, higher BMI-z score showed a two-fold greater odds for having elevated BP. Interestingly, we found that individual baseline risk factors, namely older age, Black ethnicity, lower SES and higher sugar-sweetened beverage intake significantly predicted elevated BP at follow-up, while a identified composite risk factor (including SES, family history, meat and milk product consumption) showed a significantly lower risk for elevated BP after a 4-year period.

### Individual risk factors contributing to elevated blood pressure

#### Non-modifiable risk factors in childhood and blood pressure

Chronological age, a known risk factor for elevated BP, has been found to increase the relative risk for having elevated BP over time. Although the observed association was anticipated, it remains important to highlight that even at young ages (between 5 and 9 years), increasing age already emerges as a contributing factor to elevated blood pressure. In children with rapid growth spurts, the arterial tree is still developing and adaptable, thus with growth and development, age will later become less of a risk factor and more of a time lapse indicator. Previous literature indicates that BP tends to rise linearly with chronological age [43] until plateau in late life [44]. Together with age, ethnicity and SES also play a significant role in BP status.

Socioeconomic status has previously been associated with CVD onset and progression [45]. A previous study reported that children coming from a home where the parent or guardian had a lower level of education and low income levels experienced a greater burden of cardiovascular risk [46]. Previous research also indicated that the odds of developing hypertension were 1.35-fold in adults with a lower SES compared to those with a higher SES [47]. This is in line with our study in South African children where we found that a lower SES score predicted elevated BP over time. This highlights the fact that SES is already a non-modifiable predictor of elevated BP at a young age.

Apart from having a lower SES, we also found that Black ethnicity increased the relative risk for future elevated BP. In a sensitivity analysis (*data not shown*), we found in our study population that Black children had a significantly lower SES compared to their White counterparts which may play a role in the abovementioned finding. However, our finding with regards to Black ethnicity and elevated BP is also supported by previous literature from this study cohort where the authors found that Black children had higher DBP and vascular resistance compared to their White counterparts [19]. In addition, it is also known that individuals of Black ethnicity is predisposed to a suppressed renin-angiotensin-aldosterone system [48] posing a possible physiological mechanism of why Black ethnicity may in part be a predictor of future elevated BP.

#### Modifiable risk factors in childhood and blood pressure

Higher baseline BMI-z score increased the odds of identifying elevated BP cross-sectionally. This finding is in line with current literature as there is a well-established positive association between OW/OB and BP, observed both in cross-sectional and longitudinal studies [19, 49–54]. We did not observe any predictive risk of baseline BMI-z score for elevated BP over time. Although this was unexpected, we propose that the following may have been possible reasons for the lack of associations: (i) from this seemingly healthy population, about 81% of children were classified as normal weight at baseline and the small prevalence of OW/OB may have contributed to the lack of association, (ii) the growth spurt just before puberty may be the modifying reason for the lack of these associations or (iii) children who were in the elevated BP group in baseline have undergone lifestyle behavioral changes such as higher intake of healthier food (more fruit and meat product intake) and less sugar-sweetened beverages over time following the implementation of the health promotion levy in South Africa [55]. We also observed that the children’s CRF increased which may be indicative of more regular physical activity. These factors may have played a role in the decline of elevated BP prevalence over time but could also be the reason for not seeing body composition as a predictive risk factor for elevated BP in our study population. Composite risk factor 4 composed of BMI-z score and CRF did not predict elevated BP after the four-year follow-up period, however, food intake together with SES did influence the BP status at follow-up.

Some of the main contributors to increased adiposity includes lifestyle and behavioral risk factors such as physical inactivity and excessive unhealthy food and drink intake [21, 26, 56–58], such as sugar-sweetened beverages. Sugar-sweetened beverage intake is positively associated with higher BP in several prospective studies [59–61]. To our knowledge, only a few studies investigated how sugar-sweetened beverage intake is associated with BP over time [62]. The first study, done in adults with elevated BP or hypertension at baseline, indicated that a reduction in the consumption of sugar-sweetened beverages was associated with reduced BP over 18 months [63]. Another study done in Mexican adults, also found a positive association between sugar-sweetened beverage intake and BP over a median period of 9.91 years [64]. In line with this literature, we also reported that higher intake of sugar-sweetened beverages was shown to predict elevated BP at follow-up. In our South African pediatric population, we observed that for every standard deviation increase in sugar-sweetened beverage intake, the risk for elevated BP was increased by 67%. However, upon further investigation, we observed that the intake of sugar-sweetened beverages declined between baseline and follow-up also possibly the reason for the decline in elevated BP over time. A plausible reason for the latter is the implementation of sugar taxes in April 2018 by the South African government in response to a recommendation of the World Health Organization which was three years before our follow-up measurements. A study done in adolescents, young adults and middle-aged adults living in urban Soweto indicated a decrease in consumption of sugar-sweetened beverages from before the taxation [65]. Our result supports the positive health implications proposed by the taxation on sugar and indicates that there may lie significant health benefits within the policy, even in younger consumers.

### Composite risk factor patterns and elevated blood pressure

In our study, composite risk factors did not identify any odds for having elevated BP at baseline. However, risk factor pattern 1, comprising of SES, family history, milk and meat product consumption, has shown a lower risk for elevated BP over time. Although this does not include traditional cardiovascular risk factors as expected, we can speculate that the observed lower risk for elevated BP may be attributed to healthier food intake together with higher SES. Our results indicate that higher SES is grouped together with a greater intake of protein-dense foods. Animal protein sources, particularly from meat, eggs, fish and poultry, provide essential amino acids that are vital for children’s growth and development [66]. This nutritional advantage is associated with a significantly lower risk for developing elevated BP. In contrast, as discussed earlier, we found that lower SES as an individual risk factor predicted higher longitudinal risk for elevated BP. These findings underscore the significant economic burden associated with health disparities in our country in which socioeconomic disadvantage directly contributes to adverse health trajectories. This is likely mediated by poorer dietary quality and reduced access to nutrient-rich foods, such as higher animal protein intake, which is more costly compared to refined carbohydrates. Our results further highlight the need for policies and interventions that address the social determinants of health, particularly those aimed at improving nutrition and reducing cardiovascular risk in vulnerable pediatric populations.

#### Strengths and limitations

Our findings must be interpreted within the context of its strengths and limitations. Our study included a large sample size of prepubescent children residing and attending public primary schools in the North West province of South Africa. The ExAMIN Youth SA study was well designed and followed a concise protocol in line with the European leg of this study conducted in Basel, Switzerland. This is one of the first studies in Africa reporting on a comprehensive risk factor profile in a pediatric population. In addition, this is also one of few pediatric studies in Africa to longitudinally report on pediatric BP risk factor profiles. This prospective study can report causes of elevated BP where previous cross-sectional study designs could not have reported on causal mechanisms. This study can identify BP risk without invasive, cost-effecting testing which is ideal in a pediatric population. However, this study also has several limitations. Firstly, this study cannot be seen as a conclusive representation of the general South African population as recruitment of participants only took in one educational district in the North West province of South Africa. We recommend that future studies explore other regions in South Africa to confirm whether the results remain consistent in other provinces. We also acknowledge that some of these children in our cohort might have already gone into early adolescence at the time of follow-up which may have had an influence on our results. Nonetheless, our results remain robust, as each child effectively serves as their own baseline reference, allowing for meaningful consideration of individual risk factors. Our current results remain important to report as it may demonstrate the normal course of maturation of this pediatric population within the context of their cardiovascular profile.

## Conclusion

Multiple individual and combined risk factors contributed to elevated BP risk in South African children. Multifaceted interventions, particularly dietary changes like reducing sugar-sweetened beverage intake and increasing meat and milk consumption, could curb rising BP in children. Additionally, disadvantages linked to lower SES could play a crucial role. These results emphasize the importance of demographic, socioeconomic, and dietary factors in early cardiovascular risk development, underscoring the importance of early prevention to prevent progression of CVD from childhood into adulthood.

## Supplementary information

Supplementary Material

## References

[1] Robinson CH, Chanchlani R. High blood pressure in children and adolescents: current perspectives and strategies to improve future kidney and cardiovascular health. Kidney Int Rep. 2022;7:954–70. 35570999 10.1016/j.ekir.2022.02.018 PMC9091586

[2] Falkner B, Lurbe E, Schaefer F. High blood pressure in children: clinical and health policy implications. J Clin Hypertens (Greenwich). 2010;12:261–76. 20433547 10.1111/j.1751-7176.2009.00245.x PMC8673173

[3] Juhola J, Oikonen M, Magnussen CG, Mikkila V, Siitonen N, Jokinen E, et al. Childhood physical, environmental, and genetic predictors of adult hypertension: the cardiovascular risk in young Finns study. Circulation. 2012;126:402–9. 22718800 10.1161/CIRCULATIONAHA.111.085977

[4] Song P, Zhang Y, Yu J, Zha M, Zhu Y, Rahimi K, et al. Global prevalence of hypertension in children: A systematic review and meta-analysis. JAMA Pediatr. 2019;173:1154–63. 31589252 10.1001/jamapediatrics.2019.3310 PMC6784751

[5] Oparil S, Acelajado MC, Bakris GL, Berlowitz DR, Cífková R, Dominiczak AF, et al. Hypertension. Nat Rev Dis Prim. 2018;4:18014. 29565029 10.1038/nrdp.2018.14 PMC6477925

[6] Ahn SY, Gupta C. Genetic programming of hypertension. Front Pediatr. 2017;5:285. 29404309 10.3389/fped.2017.00285 PMC5786744

[7] Falkner B, Lurbe E. Primary hypertension beginning in childhood and risk for future cardiovascular disease. J Pediatr. 2021;238:16–25. 34391765 10.1016/j.jpeds.2021.08.008

[8] Rajinikanth BS, Sujatha U, Yadav S. Prevalence of obesity and its relationship with hypertension among school-going adolescents aged 12-16 years. Cureus J Med Sci. 2023;15:e42999. 10.7759/cureus.42999 PMC10476925 37671215

[9] Craig A, Breet Y, Gafane-Matemane LF, Norris SA, Kruger R. Detecting and managing childhood onset hypertension in Africa: A call to action. Curr Hypertens Rep. 2023;25:211–30. 37318686 10.1007/s11906-023-01247-3 PMC10491553

[10] Orlando A, Cazzaniga E, Giussani M, Palestini P, Genovesi S. Hypertension in children: role of obesity, simple carbohydrates, and uric acid. Front Public Health. 2018;6:129. 29774210 10.3389/fpubh.2018.00129 PMC5943632

[11] Crouch SH, Soepnel LM, Kolkenbeck-Ruh A, Maposa I, Naidoo S, Davies J, et al. Paediatric hypertension in Africa: A systematic review and meta-analysis. EClinicalMedicine. 2022;43:101229. 34917909 10.1016/j.eclinm.2021.101229 PMC8665406

[12] Kagura J, Ong KK, Adair LS, Pettifor JM, Norris SA. Paediatric hypertension in South Africa: An underestimated problem calling for action. S Afr Med J. 2018;108:708–9. 30182892 10.7196/SAMJ.2018.v108i9.13317

[13] Su S, Wang X, Pollock JS, Treiber FA, Xu X, Snieder H, et al. Adverse childhood experiences and blood pressure trajectories from childhood to young adulthood: the Georgia stress and Heart study. Circulation. 2015;131:1674–81. 25858196 10.1161/CIRCULATIONAHA.114.013104 PMC4430378

[14] Noubiap JJ, Essouma M, Bigna JJ, Jingi AM, Aminde LN, Nansseu JR. Prevalence of elevated blood pressure in children and adolescents in Africa: a systematic review and meta-analysis. Lancet Public Health. 2017;2:e375–86. 29253478 10.1016/S2468-2667(17)30123-8

[15] Lachman JM, Cluver L, Ward CL, Hutchings J, Mlotshwa S, Wessels I, et al. Randomized controlled trial of a parenting program to reduce the risk of child maltreatment in South Africa. Child Abus Negl. 2017;72:338–51. 10.1016/j.chiabu.2017.08.014 28881303

[16] Modjadji P, Madiba S. The double burden of malnutrition in a rural health and demographic surveillance system site in South Africa: a study of primary schoolchildren and their mothers. BMC Public Health. 2019;19:1087. 31399048 10.1186/s12889-019-7412-y PMC6689169

[17] Masilela C, Pearce B, Ongole JJ, Adeniyi OV, Benjeddou M. Cross-sectional study of prevalence and determinants of uncontrolled hypertension among South African adult residents of Mkhondo municipality. BMC Public Health. 2020;20:1069. 32631300 10.1186/s12889-020-09174-7 PMC7339580

[18] Nqweniso S, Walter C, du Randt R, Aerts A, Adams L, Degen J, et al. Prevention of overweight and hypertension through cardiorespiratory fitness and extracurricular sport participation among South African schoolchildren. Sustainability. 2020;12:6581.

[19] Kruger R, Kruger HS, Monyeki MA, Pienaar AE, Roux SB, Gafane-Matemane LF, et al. A demographic approach to assess elevated blood pressure and obesity in prepubescent children: the ExAMIN Youth South Africa study. J Hypertens. 2021;39:2190–9. 34620809 10.1097/HJH.0000000000002917

[20] Sharma JR, Mabhida SE, Myers B, Apalata T, Nicol E, Benjeddou M, et al. Prevalence of hypertension and its associated risk factors in a rural black population of Mthatha Town, South Africa. Int J Environ Res Public Health. 2021;18:1215. 33572921 10.3390/ijerph18031215 PMC7908535

[21] Joubert N, Walter C, du Randt R, Aerts A, Adams L, Degen J, et al. Hypertension among South African children in disadvantaged areas and associations with physical activity, fitness, and cardiovascular risk markers: A cross-sectional study. J Sports Sci. 2021;39:2454–67. 34334121 10.1080/02640414.2021.1939964

[22] Kirschbaum TK, Sudharsanan N, Manne-Goehler J, De Neve JW, Lemp JM, Theilmann M, et al. The association of socioeconomic status with hypertension in 76 low- and middle-income countries. J Am Coll Cardiol. 2022;80:804–17. 35981824 10.1016/j.jacc.2022.05.044

[23] Lona G, Hauser C, Köchli S, Infanger D, Endes K, Schmidt-Trucksäss A, et al. Association of blood pressure, obesity and physical activity with arterial stiffness in children: a systematic review and meta-analysis. Pediatr Res. 2022;91:502–12. 33824443 10.1038/s41390-020-01278-5

[24] Köchli S, Endes K, Infanger D, Zahner L, Hanssen H. Obesity, blood pressure, and retinal vessels: A meta-analysis. Pediatrics. 2018;141:e20174090. 10.1542/peds.2017-4090 29743194

[25] Couch SC, Daniels SR. Diet and blood pressure elevation in children and adolescents. Arch Pediatr Adolesc Med. 2004;158:418–9. 15123469 10.1001/archpedi.158.5.418

[26] Kell KP, Cardel MI, Bohan Brown MM, Fernandez JR. Added sugars in the diet are positively associated with diastolic blood pressure and triglycerides in children. Am J Clin Nutr. 2014;100:46–52. 24717340 10.3945/ajcn.113.076505 PMC4144113

[27] Smith JD, Fu E, Kobayashi MA. Prevention and management of childhood obesity and its psychological and health comorbidities. Annu Rev Clin Psychol. 2020;16:351–78. 32097572 10.1146/annurev-clinpsy-100219-060201 PMC7259820

[28] Kokkinos P. Cardiorespiratory fitness, exercise, and blood pressure. Hypertension. 2014;64:1160–4. 25245388 10.1161/HYPERTENSIONAHA.114.03616

[29] Glover LM, Cain-Shields LR, Wyatt SB, Gebreab SY, Diez-Roux AV, Sims M. Life course socioeconomic status and hypertension in African American adults: The Jackson Heart Study. Am J Hypertens. 2020;33:84–91. 31420642 10.1093/ajh/hpz133 PMC6931894

[30] Edvardsson VO, Steinthorsdottir SD, Eliasdottir SB, Indridason OS, Palsson R. Birth weight and childhood blood pressure. Curr Hypertens Rep. 2012;14:596–602. 23054892 10.1007/s11906-012-0311-6

[31] Huxley RR, Shiell AW, Law CM. The role of size at birth and postnatal catch-up growth in determining systolic blood pressure: a systematic review of the literature. J Hypertens. 2000;18:815–31. 10930178 10.1097/00004872-200018070-00002

[32] Mu M, Wang SF, Sheng J, Zhao Y, Li HZ, Hu CL, et al. Birth weight and subsequent blood pressure: a meta-analysis. Arch Cardiovasc Dis. 2012;105:99–113. 22424328 10.1016/j.acvd.2011.10.006

[33] Zhou Y, Qian Z, Vaughn MG, Boutwell BB, Yang M, Zeng XW, et al. Epidemiology of elevated blood pressure and associated risk factors in Chinese children: the SNEC study. J Hum Hypertens. 2016;30:231–6. 26446390 10.1038/jhh.2015.104

[34] Jacobs JrDR, Woo JG, Sinaiko AR, Daniels SR, Ikonen J, Juonala M, et al. Childhood cardiovascular risk factors and adult cardiovascular events. N. Engl J Med. 2022;386:1877–88. 35373933 10.1056/NEJMoa2109191 PMC9563825

[35] Kruger R, Monyeki MA, Schutte AE, Smith W, Mels CMC, Kruger HS, et al. The Exercise, Arterial Modulation and Nutrition in Youth South Africa Study (ExAMIN Youth SA). Front Pediatr. 2020;8:212. 32411640 10.3389/fped.2020.00212 PMC7201091

[36] Maugana VF, Kruger R, Kruger HS, Hanssen H, Smith W. Food intake in South African children and retinal microvascular health: The ExAMIN Youth SA study. Nutr Metab Cardiovasc Dis. 2024;34:188–97. 37798229 10.1016/j.numecd.2023.08.013

[37] Kruger HS, Makore P, van Zyl T, Faber M, Ware LJ, Monyeki MA, et al. Validation of a short food group questionnaire to determine intakes from healthy and unhealthy food groups in 5-9-year-old South African children. J Hum Nutr Diet. 2024;37:234–45. 37798954 10.1111/jhn.13249 PMC10953415

[38] Ravens-Sieberer U, Bullinger M. Assessing health-related quality of life in chronically ill children with the German KINDL: first psychometric and content analytical results. Qual Life Res. 1998;7:399–407. 9691720 10.1023/a:1008853819715

[39] Deacon E, Jansen van Vuren E, Bothma E, Volschenk C, Kruger R. Validation of the parents’ version of the KINDL(R) and Kiddy Parents questionnaire in a South African context. Health Qual Life Outcomes. 2024;22:77. 39256795 10.1186/s12955-024-02292-5 PMC11389106

[40] World Health Organization. BMI-for-age: World Health Organization; 2014 [Available from: https://www.who.int/tools/growth-reference-data-for-5to19-years/indicators/bmi-for-age].

[41] Van Mechelen W, Hlobil H, Kemper H. Validation of two running tests as estimates of maximal aerobic power in children. Eur J Appl Physiol Occup Physiol. 1986;55:503–6. 3769907 10.1007/BF00421645

[42] Flynn JT, Kaelber DC, Baker-Smith CM, Blowey D, Carroll AE, Daniels SR, et al. Clinical practice guideline for screening and management of high blood pressure in children and adolescents. Pediatrics. 2017;140:e20171904. 28827377 10.1542/peds.2017-1904

[43] Chu C, Liao YY, He MJ, Ma Q, Zheng WL, Yan Y, et al. Blood Pressure Trajectories From Childhood to Youth and Arterial Stiffness in Adulthood: A 30-Year Longitudinal Follow-Up Study. Front Cardiovasc Med. 2022;9:894426. 35845038 10.3389/fcvm.2022.894426 PMC9278647

[44] Zhou B, Danaei G, Stevens GA, Bixby H, Taddei C, Carrillo-Larco RM, et al. Long-term and recent trends in hypertension awareness, treatment, and control in 12 high-income countries: an analysis of 123 nationally representative surveys. Lancet. 2019;394:639–51. 31327564 10.1016/S0140-6736(19)31145-6 PMC6717084

[45] Rosengren A, Smyth A, Rangarajan S, Ramasundarahettige C, Bangdiwala SI, AlHabib KF, et al. Socioeconomic status and risk of cardiovascular disease in 20 low-income, middle-income, and high-income countries: the Prospective Urban Rural Epidemiologic (PURE) study. Lancet Glob Health. 2019;7:e748–e760. 31028013 10.1016/S2214-109X(19)30045-2

[46] Goulding M, Goldberg R, Lemon SC. Peer reviewed: differences in blood pressure levels among children by sociodemographic status. Prev Chronic Dis. 2021;18:E88. 10.5888/pcd18.210058 PMC8462283 34529555

[47] Qin Z, Li C, Qi S, Zhou H, Wu J, Wang W, et al. Association of socioeconomic status with hypertension prevalence and control in Nanjing: a cross-sectional study. BMC Public Health. 2022;22:423. 35236306 10.1186/s12889-022-12799-5 PMC8892801

[48] Lindhorst J, Alexander N, Blignaut J, Rayner B. Differences in hypertension between blacks and whites: an overview. Cardiovasc J Afr. 2007;18:241–7. 17940670 PMC4170224

[49] Poirier P, Eckel RH. Obesity and cardiovascular disease. Curr Atheroscler Rep. 2002;4:448–53. 12361492 10.1007/s11883-002-0049-8

[50] Falkner B, Gidding SS, Ramirez-Garnica G, Wiltrout SA, West D, Rappaport EB. The relationship of body mass index and blood pressure in primary care pediatric patients. J Pediatr. 2006;148:195–200. 16492428 10.1016/j.jpeds.2005.10.030

[51] Amusa LO, Goon DT. Blood pressure among overweight children aged 7-13 years in 10 rural communities in South Africa: The Tshannda Longitudinal Study. Pak J Med Sci. 2011;27:664–7.

[52] Kang YS. Obesity associated hypertension: new insights into mechanism. Electrolyte Blood Press. 2013;11:46–52. 24627704 10.5049/EBP.2013.11.2.46 PMC3950225

[53] Ding W, Cheung WW, Mak RH. Impact of obesity on kidney function and blood pressure in children. World J Nephrol. 2015;4:223–9. 25949935 10.5527/wjn.v4.i2.223 PMC4419131

[54] Wang M, Kelishadi R, Khadilkar A, Mi Hong Y, Nawarycz T, Krzywinska-Wiewiorowska M, et al. Body mass index percentiles and elevated blood pressure among children and adolescents. J Hum Hypertens. 2020;34:319–25. 31253844 10.1038/s41371-019-0215-x

[55] Russell C, Baker P, Grimes C, Lindberg R, Lawrence MA. Global trends in added sugars and non-nutritive sweetener use in the packaged food supply: drivers and implications for public health. Public Health Nutr. 2023;26:952–64. 35899782 10.1017/S1368980022001598 PMC10346066

[56] Dumith SC, Ramires VV, Souza MA, Moraes DS, Petry FG, Oliveira ES, et al. Overweight/obesity and physical fitness among children and adolescents. J Phys Act Health. 2010;7:641–8. 20864760 10.1123/jpah.7.5.641

[57] Almuhanna MA, Alsaif M, Alsaadi M, Almajwal A. Fast food intake and prevalence of obesity in school children in Riyadh City. Sudan J Paediatr. 2014;14:71–80. 27493393 PMC4949920

[58] Agostinis-Sobrinho C, Ruiz JR, Moreira C, Abreu S, Lopes L, Oliveira-Santos J, et al. Cardiorespiratory fitness and blood pressure: A longitudinal analysis. J Pediatr. 2018;192:130–5. 29246334 10.1016/j.jpeds.2017.09.055

[59] Cheungpasitporn W, Thongprayoon C, Edmonds PJ, Srivali N, Ungprasert P, Kittanamongkolchai W, et al. Sugar and artificially sweetened soda consumption linked to hypertension: a systematic review and meta-analysis. Clin Exp Hypertens. 2015;37:587–93. 26114357 10.3109/10641963.2015.1026044

[60] Malik AH, Akram Y, Shetty S, Malik SS, Yanchou Njike V. Impact of sugar-sweetened beverages on blood pressure. Am J Cardiol. 2014;113:1574–80. 24630785 10.1016/j.amjcard.2014.01.437

[61] Cohen L, Curhan G, Forman J. Association of sweetened beverage intake with incident hypertension. J Gen Intern Med. 2012;27:1127–34. 22539069 10.1007/s11606-012-2069-6 PMC3515007

[62] Kruger HS, van Zyl T, Monyeki MA, Ricci C, Kruger R. Decreased frequency of sugar-sweetened beverages intake among young children following the implementation of the health promotion levy in South Africa. Public Health Nutr. 2025;28:e23. 39764638 10.1017/S1368980024002623 PMC11822614

[63] Chen L, Caballero B, Mitchell DC, Loria C, Lin P-H, Champagne CM, et al. Reducing consumption of sugar-sweetened beverages is associated with reduced blood pressure: a prospective study among United States adults. Circulation. 2010;121:2398–406. 20497980 10.1161/CIRCULATIONAHA.109.911164 PMC2892032

[64] Hernández-López R, Canto-Osorio F, Vidaña-Pérez D, Torres-Ibarra L, Rivera-Paredez B, Gallegos-Carrillo K, et al. Soft drink and non-caloric soft drink intake and their association with blood pressure: the Health Workers Cohort Study. Nutr J. 2022;21:37. 35668525 10.1186/s12937-022-00792-y PMC9171938

[65] Wrottesley SV, Stacey N, Mukoma G, Hofman KJ, Norris SA. Assessing sugar-sweetened beverage intakes, added sugar intakes and BMI before and after the implementation of a sugar-sweetened beverage tax in South Africa. Public Health Nutr. 2021;24:2900–10. 33315006 10.1017/S1368980020005078 PMC9884749

[66] Zaharia S, Ghosh S, Shrestha R, Manohar S, Thorne-Lyman AL, Bashaasha B, et al. Sustained intake of animal-sourced foods is associated with less stunting in young children. Nat Food. 2021;2:246–54. 37118465 10.1038/s43016-021-00259-z

