# Supplementary material for: A prospective analysis to assess the multifactorial risk of childhood-onset hypertension: the ExAMIN Youth SA study
Source: Hypertens Res. 2025 Aug 15;48(10):2618–30. doi: 10.1038/s41440-025-02309-6 (PMC12497645; doi:10.1038/s41440-025-02309-6)
Supplement: Supplementary file 1 — Supplementary Material [file 41440_2025_2309_MOESM1_ESM.docx]

**Table S1: Baseline characteristics of the ExAMIN Youth SA population stratified by blood pressure status**

|  | **Normal blood pressure**  (n = 484) | **Elevated blood pressure**  (n = 283) | **p-value** |
| --- | --- | --- | --- |
| **Non-modifiable risk factors** | | | |
| Age, years | 7.39 ± 0.94 | 7.50 ± 0.86 | 0.11 |
| Sex, girls, *n (%)* | 277 (57.2) | 151 (53.4) | 0.30 |
| Ethnicity, Black, *n (%)* | 286 (59.1) | 167 (59.0) | 0.98 |
| Socioeconomic status | 5.53 ± 2.66 | 5.93 ± 2.58 | 0.069 |
| Health-related quality of life | 61.03 ± 7.78 | 61.09 ± 7.44 | 0.93 |
| Family history, yes, n (%) | 68 (38.6) | 49 (46.2) | 0.21 |
| **Modifiable risk factors** | | | |
| Systolic blood pressure, mmHg | 97 (96; 97) | 110 (110; 111) | **<0.001** |
| Diastolic blood pressure, mmHg | 61 (60; 62) | 70 (69; 70) | **<0.001** |
| Mean arterial pressure, mmHg | 76 (75; 76) | 86 (86; 87) | **<0.001** |
| ***Body composition*** |  |  |  |
| Body weight, kg | 23.9 ± 5.44 | 26.5 ± 7.43 | **<0.001** |
| Body height, m | 122.1 ± 7.69 | 123.8 ± 7.81 | **0.006** |
| BMI z-score | -0.17 ± 1.10 | 0.32 ± 1.15 | **<0.001** |
| Overweight/Obesity status, *n (%)* | 57 (11.8) | 85 (30.0) | **<0.001** |
| ***Cardiorespiratory fitness*** | | | |
| 20-m shuttle runs | 26.7 ± 13.5 | 27.8 ± 12.1 | 0.39 |
| Heart rate, bpm | 86 ± 11 | 89 ± 12 | **<0.001** |
| ***Food intake frequency per week*** |  |  |  |
| Fruits | 3.21 ± 2.15 | 3.30 ± 2.15 | 0.62 |
| Vegetables | 3.04 ± 1.90 | 2.84 ± 1.97 | 0.18 |
| Milk, yogurt | 4.33 ± 2.37 | **4.16** ± 2.44 | 0.38 |
| Meat, fish, poultry, eggs | 4.80 ± 2.09 | 4.68 ± 2.11 | 0.50 |
| Cold sweetened and milk drinks | 4.08 ± 2.49 | 4.08 ± 2.45 | 0.97 |
| Tea and hot drinks with sugar | 3.26 ± 2.70 | 3.13 ± 2.70 | 0.55 |
| Sweets | 2.83 ± 2.05 | 2.79 ± 2.16 | 0.80 |
| Salty snacks | 2.86 ± 2.15 | 3.02 ± 2.26 | 0.35 |
| Cookies, cakes, biscuits | 2.12 ± 1.80 | 2.01 ± 1.76 | 0.42 |
| Fast foods | 1.71 ± 1.58 | 1.69 ± 1.50 | 0.88 |
| ***Urinary biomarkers*** |  |  |  |
| Sodium | 16.3 ± 1.80 | 16.6 ± 1.81 | 0.66 |
| Potassium | 3.47 ± 2.00 | 3.24 ± 2.02 | 0.21 |
| Sodium/Potassium ratio | 0.59 ± 2.40 | 0.62 ± 2.37 | 0.34 |
| Values are reported as mean ± standard deviation, number (percentage) or mean and 95% confidence intervals. Values were obtained using independent T-tests, Chi-square tests and ANCOVA for blood pressure adjusting for age sex and height. Urinary biomarkers were corrected for urinary creatinine. Data in boldface indicate statistical significance for p≤ 0.05. | | | |

**Table S2: Multiple regression analyses of risk factors with blood pressure in the total population and blood pressure groups at baseline**

|  | **Systolic blood pressure** | | |  | **Diastolic blood pressure** | |
| --- | --- | --- | --- | --- | --- | --- |
| **Total group (N=767)** | | | | | | |
|  | Adj R^2^ = 0.143 | |  | | Adj R^2^ = 0.085 | |
|  | Std β (95% CI) | p |  | | Std β (95% CI) | p |
| Age | 0.18 (0.037; 0.32) | **0.014** | Age | | ─ | ─ |
| BMI-z | 0.29 (0.16; 0.41) | **<0.001** | BMI-z | | 0.20 (0.073; 0.33) | **0.002** |
|  |  |  | Family history | | 0.41 (0.11; 0.70) | **0.008** |
| **Normotensive group (n=484)** | | | | | | |
|  | Adj R^2^ = 0.100 | |  | | Adj R^2^ = 0.080 | |
|  | Std β (95% CI) | p |  | | Std β (95% CI) | p |
| Age | 0.14 (0.015; 0.26) | **0.028** | Age | | 0.13 (0.006; 0.26) | **0.040** |
| BMI-z | 0.16 (0.048; 0.28) | **0.006** | BMI-z | | 0.11 (-0.004; 0.50) | 0.058 |
|  |  |  | Family history | | 0.23 (-0.028; 0.50) | 0.079 |
| **Elevated BP group (n=283)** | | | | | | |
|  | Adj R^2^ = 0.125 | |  | | Adj R^2^ = NS | |
|  | Std β (95% CI) | p |  | | Std β (95% CI) | p |
| Age | 0.20 (-0.014; 0.41) | 0.067 | ─ | | ─ | ─ |
| BMI-z | 0.19 (0.014; 0.36) | **0.034** | ─ | | ─ | ─ |

Backward stepwise multiple regression analyses were used obtain p-values. Data in boldface indicate statistical significance for p≤ 0.05. Abbreviations: BP, blood pressure; CI, confidence intervals.

**Table S3: Probability of baseline individual risk factors associating with elevated blood pressure in children at baseline**

|  | **Odds ratio** | **95% CI** | **p-value** |
| --- | --- | --- | --- |
| **Nagelkerke R^2^ = 0.14** | | | |
| BMI-z | 2.21 | 1.37; 3.55 | **0.001** |

Odds ratio and 95% confidence intervals (CI) of a one standard deviation increase in risk factors for elevated blood pressure. Data in boldface indicates statistical significance for p≤ 0.05.

**Table S4: Probability of baseline risk factors in children predicting elevated blood pressure over a four-year-follow-up period**

|  | **Hazard ratio** | **95% CI** | **p-value** |
| --- | --- | --- | --- |
| Age | 1.78 | 1.19; 2.66 | **0.005** |
| Ethnicity | 0.048 | 0.007; 0.31 | **0.001** |
| Socioeconomic status | 0.42 | 0.24; 0.76 | **0.004** |
| Fruits | 1.59 | 0.99; 2.53 | 0.054 |
| Sugar sweetened beverages | 1.67 | 1.07; 2.61 | **0.026** |
| Baseline blood pressure category | 0.53 | 0.26; 1.08 | 0.078 |

Hazard ratio and 95% confidence intervals (CI) of a one standard deviation increase in risk factors for elevated blood pressure. Data in boldface indicate statistical significance for p≤ 0.05. Adjusted for baseline blood pressure.

**Table S5: Factor patterns identified in the total population of children aged between five and nine years**

| **Total population** | | | | | |
| --- | --- | --- | --- | --- | --- |
|  | **Pattern 1** | **Pattern 2** | **Pattern 3** | **Pattern 4** | **Pattern 5** |
| Socioeconomic status | 0.76 | ─ | ─ | ─ | ─ |
| Family history | -0.65 | ─ | ─ | ─ | ─ |
| Milk | 0.65 | ─ | ─ | ─ | ─ |
| Meat | 0.62 | ─ | ─ | ─ | ─ |
| Chips | ─ | 0.73 | ─ | ─ | ─ |
| Sweets | ─ | 0.71 | ─ | ─ | ─ |
| Cookies and cake | ─ | 0.64 | ─ | ─ | ─ |
| Fast foods | ─ | 0.55 | ─ | ─ | ─ |
| Vegetables | ─ | ─ | 0.75 | ─ | ─ |
| Fruits | ─ | ─ | 0.76 | ─ | ─ |
| Pacer laps | ─ | ─ | ─ | -0.78 | ─ |
| BMI-z score | ─ | ─ | ─ | 0.56 | ─ |
| Quality of life | ─ | ─ | ─ | ─ | 0.68 |
| Sodium/Potassium ratio | ─ | ─ | ─ | ─ | 0.63 |
| Sugar hot drinks | ─ | ─ | ─ | ─ | ─ |
| Sugar-sweetened beverages | ─ | ─ | ─ | ─ | ─ |
| Principal component analyses were used to obtain factor patterns. | | | | | |

**Table S6: Multiple regression analyses of the identified factor patterns with blood pressure in the total population and stratified by blood pressure status**

|  | **Systolic blood pressure** | | |  | **Diastolic blood pressure** | | |
| --- | --- | --- | --- | --- | --- | --- | --- |
| **Total group** | | | | | | | |
|  | **Adj R^2^** | **Std β (95% CI)** | **p-value** |  | **Adj R^2^** | **Std β (95% CI)** | **p-value** |
| ***Factor 1*** | 0.056 | ─ | ─ | ***Factor 1*** | 0.038 | -0.16 (-0.28; -0.035) | **0.012** |
| Age |  | 0.22 (0.10; 0.34) | **<0.001** | Age |  | 0.13 (0.008; 0.25) | **0.037** |
| Sex |  | ─ | ─ | Sex |  | ─ | ─ |
| Ethnicity |  | 0.26 (0.015; 0.50) | **0.037** | Ethnicity |  | ─ | ─ |
| ***Factor 2*** | 0.061 | ─ | ─ | ***Factor 2*** | 0.026 | ─ | ─ |
| Age |  | 0.22 (0.15; 0.30) | **<0.001** | Age |  | 0.14 (0.068; 0.22) | **<0.001** |
| Sex |  | ─ | ─ | Sex |  | ─ | ─ |
| Ethnicity |  | 0.26 (0.11; 0.41) | **<0.001** | Ethnicity |  | -0.18 (-0.33; -0.020) | **0.027** |
| ***Factor 3*** | 0.061 | ─ | ─ | ***Factor 3*** | 0.029 | ─ | ─ |
| Age |  | 0.22 (0.15; 0.30) | **<0.001** | Age |  | 0.14 (0.070; 0.22) | **<0.001** |
| Sex |  | ─ | ─ | Sex |  | -0.13 (-0.28; 0.024) | 0.099 |
| Ethnicity |  | 0.26 (0.11; 0.41) | **<0.001** | Ethnicity |  | -0.16 (-0.31; -0.006) | **0.042** |
| ***Factor 4*** | 0.078 | -0.15 (-0.24; -0.057) | **0.001** | ***Factor 4*** | 0.032 | -0.097 (-0.19; -0.004) | **0.040** |
| Age |  | 0.25 (0.16; 0.34) | **<0.001** | Age |  | 0.16 (0.072; 0.26) | **0.072** |
| Sex |  | ─ | ─ | Sex |  | ─ | ─ |
| Ethnicity |  | 0.22 (0.038; 0.40) | **0.017** | Ethnicity |  | -0.20 (-0.39; -0.020) | **0.030** |
| ***Factor 5*** | 0.060 | ─ | ─ | ***Factor 5*** | 0.025 | ─ | ─ |
| Age |  | 0.22 (0.14; 0.31) | **<0.001** | Age |  | 0.14 (0.057; 0.23) | **0.001** |
| Sex |  | ─ | ─ | Sex |  | ─ | ─ |
| Ethnicity |  | 0.26 (0.086; 0.43) | **0.003** | Ethnicity |  | -0.18 (-0.35; 0.000) | **0.050** |
| **Normal blood pressure** | | | | | | | |
|  | **Adj R^2^** | **Std β (95% CI)** | **p-value** |  | **Adj R^2^** | **Std β (95% CI)** | **p-value** |
| ***Factor 1*** | 0.083 | -0.16 (-0.31; -0.014) | **0.032** | ***Factor 1*** | 0.057 | -0.11 (-0.21; 0.003) | 0.057 |
| Age |  | 0.15 (0.046; 0.26) | **0.005** | Age |  | 0.14 (0.031; 0.24) | **0.012** |
| Sex |  | ─ | ─ | Sex |  | ─ | ─ |
| Ethnicity |  | 0.44 (0.14; 0.73) | **0.004** | Ethnicity |  | ─ | ─ |
| ***Factor 2*** | 0.069 | ─ | ─ | ***Factor 2*** | 0.048 | ─ | ─ |
| Age |  | 0.16 (0.094; 0.22) | **<0.001** | Age |  | 0.15 (0.081; 0.21) | **0.081** |
| Sex |  | ─ | ─ | Sex |  | -0.12 (-0.25; 0.020) | 0.094 |
| Ethnicity |  | 0.22 (0.086; 0.36) | **0.001** | Ethnicity |  | ─ | ─ |
| ***Factor 3*** | 0.069 | ─ | ─ | ***Factor 3*** | 0.048 | ─ | ─ |
| Age |  | 0.16 (0.096; 0.22) | **<0.001** | Age |  | 0.15 (0.082; 0.21) | **<0.001** |
| Sex |  | ─ | ─ | Sex |  | -0.12 (-0.25; 0.017) | 0.088 |
| Ethnicity |  | 0.22 (0.089; 0.35) | **0.001** | Ethnicity |  | ─ | ─ |
| ***Factor 4*** | 0.10 | -0.14 (-0.21; -0.058) | **<0.001** | ***Factor 4*** | 0.077 | -0.14 (-0.21; -0.060) | **<0.001** |
| Age |  | 0.19 (0.12; 0.26) | **<0.001** | Age |  | 0.18 (0.10; 0.25) | **<0.001** |
| Sex |  |  |  | Sex | ─ | ─ | ─ |
| Ethnicity |  | 0.20 (0.053; 0.36) | **0.008** | Ethnicity | ─ | ─ | ─ |
| ***Factor 5*** | 0.067 | ─ | ─ | ***Factor 5*** | 0.043 | ─ | ─ |
| Age |  | 0.16 (0.085; 0.23) | **<0.001** | Age |  | 0.15 (0.073; 0.22) | **<0.001** |
| Sex |  |  |  | Sex |  | ─ | ─ |
| Ethnicity |  | 0.22 (0.067; 0.38) | **0.005** | Ethnicity |  | ─ | ─ |
| **Elevated blood pressure** | | | | | | | |
|  | **Adj R^2^** | **Std β (95% CI)** | **p-value** |  | **Adj R^2^** | **Std β (95% CI)** | **p-value** |
| ***Factor 1*** | 0.10 | ─ | ─ | ***Factor 1*** | 0.049 | -0.24 (-0.43; -0.047) | **0.015** |
| Age |  | 0.23 (0.078; 0.39) | **0.004** | Age |  | ─ | ─ |
| Sex |  |  |  | Sex |  | ─ | ─ |
| Ethnicity |  | 0.31 (0.007; 0.61) | **0.045** | Ethnicity |  | ─ | ─ |
| ***Factor 2*** | 0.11 |  |  | ***Factor 2*** | 0.054 | ─ | ─ |
| Age |  | 0.23 (0.14; 0.33) | **<0.001** | Age |  | ─ | ─ |
| Sex |  | ─ | ─ | Sex |  | -0.26 (-0.50; -0.012) | **0.040** |
| Ethnicity |  | 0.31 (0.12; 0.50) | **0.002** | Ethnicity |  | -0.39 (-0.64; -0.14) | **0.002** |
| ***Factor 3*** | 0.11 |  |  | ***Factor 3*** | 0.054 | ─ | ─ |
| Age |  | 0.23 (0.14; 0.33) | **<0.001** | Age |  | ─ | ─ |
| Sex |  | ─ | ─ | Sex |  | -0.26 (-0.50; -0.018) | **0.035** |
| Ethnicity |  | 0.31 (0.12; 0.49) | **0.001** | Ethnicity |  | -0.39 (-0.63; -0.15) | **0.002** |
| ***Factor 4*** | 0.13 | -0.14 (-0.26; -0.018) | **0.025** | ***Factor 4*** | 0.050 | ─ | ─ |
| Age |  | 0.26 (0.13; 0.38) | **<0.001** | Age |  | ─ | ─ |
| Sex |  | ─ | ─ | Sex |  | -0.26 (-0.56; 0.048) | 0.098 |
| Ethnicity |  | 0.22 (-0.020; 0.47) | 0.072 | Ethnicity |  | -0.39 (-0.70; -0.080) | **0.014** |
| ***Factor 5*** | 0.11 |  |  | ***Factor 5*** | 0.052 | ─ | ─ |
| Age |  | 0.23 (0.12; 0.35) | **<0.001** | Age |  | ─ | ─ |
| Sex |  | ─ | ─ | Sex |  | -0.26 (-0.54; 0.019) | 0.067 |
| Ethnicity |  | 0.31 (0.093; 0.52) | **0.005** | Ethnicity |  | -0.39 (-0.67; -0.11) | **0.007** |

Multiple regression analyses were used to obtain p-values. Data in boldface indicate statistical significance for p≤0.050. Factors were separately analyzed due to collinearity between some of the factors.

**Table S7: Probability of the identified factor patterns predicting elevated blood pressure in children at baseline**

|  | **Nagelkerke R^2^** | **Odds ratio** | **95% CI** | **p-value** |
| --- | --- | --- | --- | --- |
| Factor 1 | 0.013 | 1.16 | 0.83; 1.16 | 0.38 |
| Factor 2 | 0.012 | 0.98 | 0.83; 1.16 | 0.84 |
| Factor 3 | 0.011 | 1.02 | 0.87; 1.19 | 0.81 |
| Factor 4 | 0.007 | 0.98 | 0.80; 1.20 | 0.98 |
| Factor 5 | 0.032 | 1.0 | 0.82; 1.21 | 0.99 |
| Logistic regression analyses were used to obtain values. Each factor was analyzed in a separate model due to collinearity of factors. Models consisted of age, sex, ethnicity, and a factor. | | | | |
